# A Simple Approach to Achieving Ultrasmall III-Nitride Microlight-Emitting Diodes with Red Emission

**DOI:** 10.1021/acsaelm.2c00311

**Published:** 2022-05-18

**Authors:** Peng Feng, Ce Xu, Jie Bai, Chenqi Zhu, Ian Farrer, Guillem Martinez de Arriba, Tao Wang

**Affiliations:** Department of Electronic and Electrical Engineering, The University of Sheffield, Sheffield S1 3JD, United Kingdom

**Keywords:** InGaN, microLED, selective epitaxy growth, patterned template, MOVPE, EQE

## Abstract

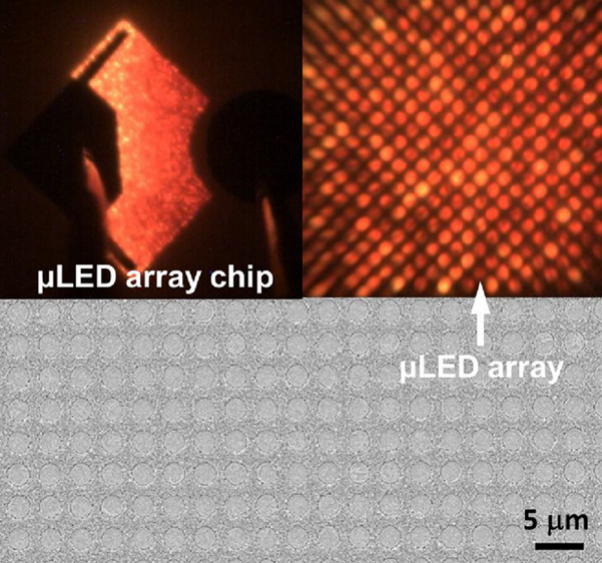

The microdisplays for augmented reality and virtual reality require ultrasmall micro light-emitting-diodes (μLEDs) with a dimension of ≤5 μm. Furthermore, the microdisplays also need three kinds of such μLEDs each emitting red, green, and blue emission. Currently, in addition to a great challenge for achieving ultrasmall μLEDs mainly based on III-nitride semiconductors, another fundamental barrier is due to an extreme difficulty in growing III-nitride-based red LEDs. So far, there has not been any effective approach to obtain high indium content InGaN as an active region required for a red LED while maintaining high optical performance. In this paper, we have demonstrated a selective epitaxy growth approach using a template featuring microhole arrays. This allows us to not only obtain the natural formation of ultrasmall μLEDs but also achieve InGaN with enhanced indium content at an elevated growth temperature, at which it is impossible to obtain InGaN-based red LEDs on a standard planar surface. By means of this approach, we have demonstrated red μLEDs (at an emission wavelength of 642 nm) with a dimension of 2 μm, exhibiting a high luminance of 3.5 × 10^7^ cd/m^2^ and a peak external quantum efficiency of 1.75% measured in a wafer form (i.e., without any packaging to enhance an extraction efficiency). In contrast, an LED grown under identical growth conditions but on a standard planar surface shows green emission at 538 nm. This highlights that our approach provides a simple solution that can address the two major challenges mentioned above.

## 1 Introduction

There is a growing interest for developing microdisplays with compact screens of ≤1/4″ diagonal length, which have a wide range of applications in smart watches, smart phones, smart bands, and augmented reality and virtual reality (AR & VR) devices.^1−5^ Their individual pixel elements typically consist of a large number of microscale visible LEDs mainly based on III-nitride semiconductors, which are referred to as microLEDs (μLEDs). For instance, the microdisplays for AR and VR require μLEDs with an ultrasmall dimension of ≤5 μm.^6−8^ Such devices are typically utilized in a scenario where spaces are small or the devices need to be close to the eyes. Therefore, the devices require high resolution, high contrast ratio, high luminance, and high external quantum efficiency (EQE).^9,10^ Of course, a microdisplay needs three kinds of individual μLEDs as a single pixel each emitting red, green, and blue emission (i.e., RGB), respectively.

InGaN semiconductors have direct bandgaps across their entire composition ranging from 0.7 eV for InN to 3.43 eV for GaN, covering part of the infrared region, the full visible spectrum, and part of the ultraviolet (UV) region. So far, InGaN-based μLEDs with reasonably good performance in the blue and green spectral region have been reported. However, red LEDs still rely on AlGaInP materials. Although a large area AlGaInP red LED with a high efficiency of >50% can be obtained,^11^ the efficiency reduces dramatically when its dimension is reduced to the microscale, namely, μLEDs. This is due to an enhancement in the surface recombination rate and the long carrier diffusion lengths.^12−15^ Moreover, the efficiency of AlGaInP red LEDs is sensitive to their junction temperature,^16,17^ and thus, AlGaInP red LEDs generally suffer from a severe leakage current at a high temperature, generating a severe efficiency thermal drop. All these fundamental issues indicate that it is indispensable to develop III-nitride based red LEDs to meet the requirements for the fabrication of a full color microdisplay.

InGaN with high indium content (>20%) is necessary for obtaining long wavelength emission. Unfortunately, it is greatly challenging to achieve high indium content InGaN while maintaining high optical performance.^18,19^ A typical method to achieve high indium content in InGaN is to lower the growth temperature for InGaN. However, it is clear that this method is not ideal because it causes a significant degradation in crystal quality.

In general, vapor–solid thermodynamic equilibrium can be modified by stress, making the solid-phase epitaxial composition reduce toward lattice-matched conditions. This is the major reason why it is so difficult to increase indium incorporation into GaN.^20−24^ Therefore, the growth of InGaN on a relaxed layer is beneficial for obtaining high indium content in InGaN. However, bear in mind that the formation of a relaxed layer is often associated with the generation of extra defects if a heterostructure with a large lattice mismatch is used to generate a relaxed layer. This leads to degradation in optical performance. Furthermore, the stress status of an underlying layer plays a critical role in determining indium incorporation into GaN. Generally speaking, tensile stress tends to enhance indium incorporation into GaN, offering a unique advantage for growing red LEDs on silicon substrates as GaN on Si suffering tensile stress.^25,26^ In contrast, GaN grown on sapphire substrates exhibits compressive strain.

The growth of InGaN-based red LEDs has been reported by means of inserting a thin AlN or an AlGaN layer into each InGaN quantum well as an emitting region, leading to an enhancement in strain that pushes the emission wavelength of InGaN quantum wells toward longer wavelength.^27,28^ So far, this approach has become a popular method for the growth of long wavelength emitters, in particular, red LEDs.^25−30^ Furthermore, by combining the idea of inserting a thin AlN or AlGaN and further adjusting the in-plane strain of a GaN template by tuning the GaN thickness, 633 nm-wavelength red LEDs with an external quantum efficiency (EQE) of 1.6% has been reported, where an extremely thick GaN (8–10 μm) template has been employed.^26,29^ A more recent report has demonstrated that a peak EQE as high as 4.5% has been achieved on red InGaN μLEDs but with a large dimnsion (60 × 60 μm^2^).^31^ However, it is worth noting that the approach based on enhanced strain also leads to a reduction in internal quantum efficiency.

We expect that an enhanced relaxation can be achieved by using selective epitaxy growth on a microhole-patterned template, which we have developed recently, where μLEDs can be naturally formed but without employing any dry-etching techniques because selective epitaxy growth can take place only within these microholes.^7,8^ In this work, we are proposing to employ this approach to achieve ultrasmall red μLED arrays with enhanced quantum efficiency but without inserting any thin AlN or AlGaN into InGaN quantum wells as an emitting region. It is expected that no lateral confinement during the selective epitaxy growth process leads to strain relaxation effectively and naturally. By this mechanism, 642 nm red μLEDs with a dimension of 2 μm have been achieved by our selective epitaxy growth conducted at an elevated temperature, at which a red LED cannot be achieved on a standard planar GaN surface. The resultant external quantum efficiency is 1.75%. For comparison, only 538 nm green LEDs on a standard planar GaN template can be obtained even under identical growth conditions. Our X-ray diffraction measurements have confirmed that a significant enhancement in indium content in InGaN has been achieved by our approach.

## 2 Results and Discussion

In this work, two different InGaN-based LED samples have been designed and then grown, aiming to study the influence of selective epitaxial growth on the optical performance of III-nitride LEDs grown on a pre-patterned template featuring microhole arrays. A μLED array sample is obtained by our selective epitaxy growth on the pre-patterned n-GaN template as mentioned above and is denoted as LED A. The other one is a normal LED sample grown under identical growth but on a standard planar n-GaN template without any features and is denoted as LED B.

Silicon-doped n-GaN epiwafers are first grown on *c*-plane (0001) sapphire substrates using the standard two-step approach by a metalorganic vapor phase epitaxy (MOVPE) technique. Initially, a 25 nm GaN nucleation layer is prepared at a low temperature after the substrate is subject to a thermally annealing process at a high temperature of 1150 °C, followed by a 1 μm GaN buffer layer, and then another 500 nm silicon-doped n-GaN layer both grown at a high temperature of 1120 °C. For LED A, the n-GaN template is further patterned into microhole arrays using SiO_2_ masks on its top, which is then used as a pre-patterned template for our selective epitaxial growth.

**Figure 1:**
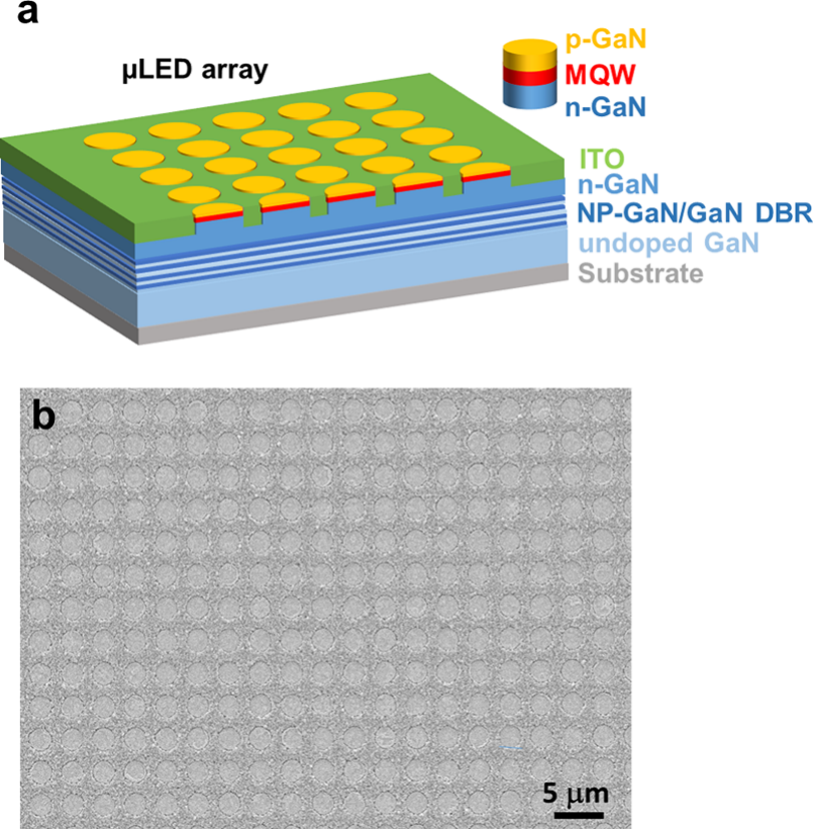
(a) Schematic of selective epitaxy growth and (b) plan-view SEM image for the μLED array epiwafer, showing a diameter of 2 μm and an interpitch of 1.5 μm.

Figure 1a shows the schematics of our selective epitaxy growth approach, allowing us to naturally achieve μLED arrays without involving any dry-etching process, i.e., LED A. For the detailed information on fabricating the prepatterned templates, refer to the Experimental Methods section.

Afterward, a standard III-nitride LED structure is selectively grown on the micropatterned template by MOVPE, namely, a silicon-doped n-GaN layer is first prepared, followed by an In_0.05_Ga_0.95_N/GaN superlattice (SLS) structure as a prelayer, five periods of InGaN/GaN multiple quantum wells (MQWs) as an emitting region, then a 20 nm *p*-type Al_0.2_Ga_0.8_N as an electron blocking layer, and a final 150 nm *p*-type GaN layer. The total thickness of the overgrown layers is 500 nm, which matches the thickness of the SiO_2_ masks. Due to the SiO_2_ masks, the growth of the LED structure takes place within the microholes only, naturally forming regularly arrayed μLEDs.

A Raith 150 scanning electron microscopy (SEM) system has been used to characterize the surface morphology of our regularly arrayed μLEDs. Figure 1b shows a typical plan-view SEM image of our regularly arrayed μLEDs wafer (i.e., LED A), exhibiting a nice circular shape with an excellent high uniformity in shape, diameter, and interpitch. All μLEDs are 2 μm in diameter and only 1.5 μm in interpitch. Such a small diameter and an interpitch are crucial for manufacturing a high-resolution microdisplay in a compact manner. Furthermore, the μLED pixels share a common *n* contact while all the *p* contacts are left open. As a result, our regularly arrayed μLED epiwafers well-match any existing manufacturing technique of microdisplays, for instance, the pick-and-place technology, which has been widely used,^32^ and the integrating technique using driving transistors based on the silicon CMOS IC to achieve individually addressable μLED-based microdisplays.^33^

**Figure 2:**
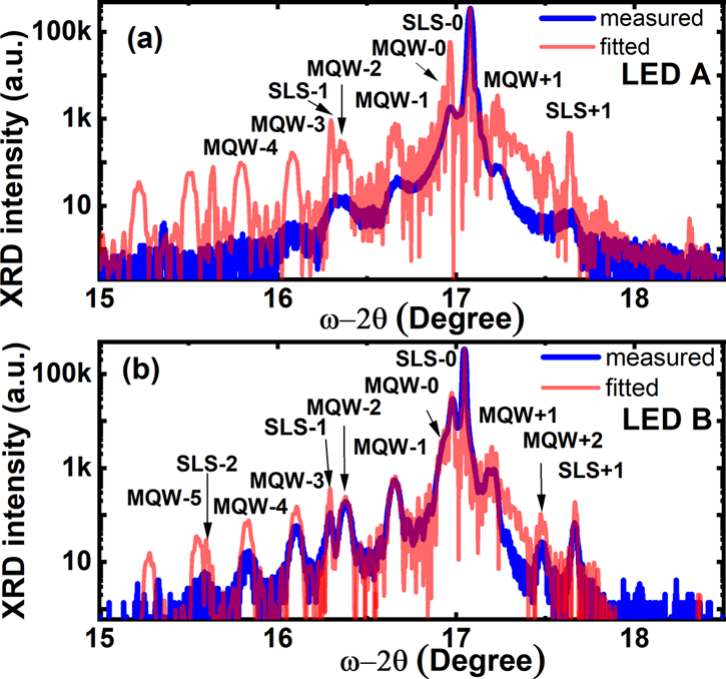
HRXRD ω-2θ scan curves of the μLED array sample on a patterned template, i.e., (a) LED A, and the LED sample grown on a standard planar template, i.e., (b) LED B under identical conditions. Fittings have also been provided to determine the indium content in InGaN MQWs.

A high-resolution X-ray diffractometer (HRXRD) (Bruker D8) has been employed to determine the indium content of the InGaN MQWs by performing ω-2θ scan measurements along the (002) direction, together with a fitting using the Bruker JV-RADS simulation software. Figure 2a,b shows the HRXRD ω-2θ scan curves of our regularly arrayed μLED wafer (i.e., LED A) and the standard LED wafer (i.e., LED B), respectively. In both cases, the satellite peaks with up to 4 or 5 orders from the InGaN/GaN MQWs have been clearly observed. The satellite peaks from the SLS structure as a prelayer have also been observed. Based on a detailed fitting, it can be determined that the indium content of the InGaN MQWs of LED A is 31% and that the thicknesses of the InGaN quantum well and the barrier are 2.2 and 13.8 nm, respectively. In contrast, LED B exhibits 24% indium content in the InGaN MQWs with a 2.6 nm quantum well and a 14.1 nm barrier. The XRD fittings are conducted based on fully strained InGaN MQWs for both LEDs. It is well known that strain relaxation will reduce the strain-induced quantum-confined Stark effect (QCSE), leading to a blue-shift in the emission. It means that if the InGaN MQWs are assumed to be strain-relaxed, the indium content should be even higher. In consideration of a higher chance of strain relaxation for the μLEDs, the fitted values of indium contents represent the least difference between the two LEDs. This direct comparison indicates that enhanced indium content in InGaN MQWs can be obtained by using our selective epitaxy growth approach on a prepatterned template featuring microhole arrays.

Finally, both the regularly arrayed μLED wafer (i.e., LED A) and the standard LED wafer (i.e., LED B) have been fabricated into LED devices with an area of 330 × 330 μm^2^. For the detailed information about device fabrication, refer to the Experimental Methods section. For LED A, each LED device consists of a few thousands of 2 μm μLEDs connected. In this work, the μLEDs in LED A share a common *p* contact and *n* contact, which are driven simultaneously in all electroluminescence (EL) measurements. However, it is worth noting that our arrayed μLEDs are designed to make the *p* contacts of each μLED left open, providing an opportunity in the future to allow indium bumps to be bonded to an active matrix driving transistors. This means that our regularly arrayed μLED structure entirely matches any existing approach for the fabrication of individually addressable μLED microdisplays.

For a direct comparison, the LED B wafer has also been processed under identical conditions in the same batch. All the characteristics of our μLED chips in the present study have been carried out on bare chips, meaning that we did not use coating or passivation or epoxy or reflector for improving extraction efficiency. Current–voltage (*I*–*V*) characteristic and EL measurements have been performed at room temperature in a continuous wave (CW) mode using a Keithley 2400 sourcemeter on a probe station equipped with an optical microscopy system.

**Figure 3:**
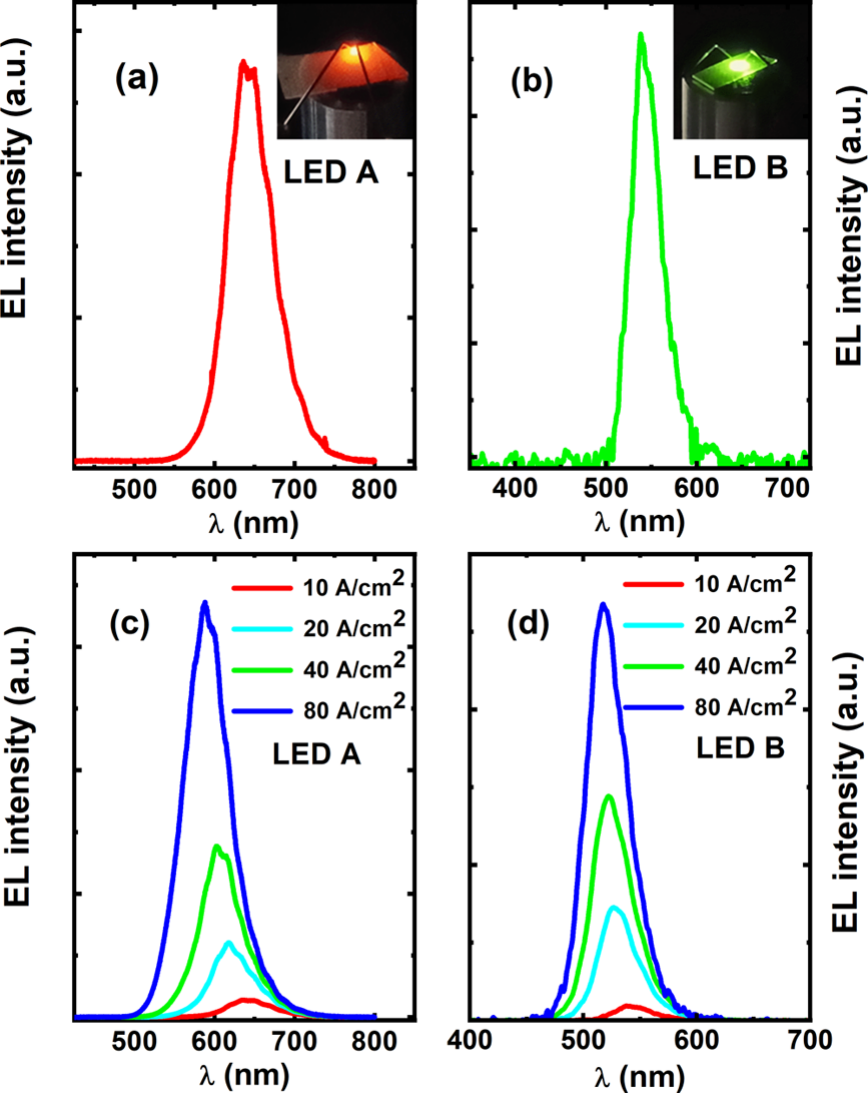
EL spectra measured at 10 A/cm^2^ for the μLED array device, i.e., (a) LED A and (b) LED B, where the insets show their respective emission images. EL spectra measured at increased current densities from 10 to 80 A/cm^2^ for (c) LED A and (d) LED B, respectively.

The EL spectra have been measured on the two LED devices under identical conditions aiming to make a direct comparison. For instance, Figure 3a,b shows the EL spectra of the two LED devices measured at a current density of 10 A/cm^2^, respectively. Both spectra exhibit a single emission peak. The μLED array device shows a strong emission at an emission wavelength of 642 nm in the red spectral region. The inset of Figure 3a exhibits an emission image of the μLED array chip, demonstrating red light. In contrast, Figure 3b displays a strong green emission at 538 nm from the LED B device also measured at 10 A/cm^2^, and the inset displays its emission image. This means that the selective epitaxial growth on a pre-patterned template featuring regularly arrayed microholes results in a red-shift of about 100 nm in emission wavelength in comparison with the LED grown on a standard planar GaN surface, although both are grown under identical growth conditions. As discussed earlier, the growth of InGaN on a relaxed layer is beneficial for obtaining high indium content in InGaN. Due to the fact that there is no lateral confinement during the overgrowth within the microholes, the overgrown n-GaN is very likely strain-relaxed, which leads to an enhancement in the indium content in the overlying InGaN MQWs. Combined with the XRD results, it has been confirmed that our selective epitaxy growth approach can enhance indium incorporation into GaN significantly. Figure 3c,d shows the EL spectra of LED A and LED B, both measured as a function of injection current density ranging from 10 to 80 A/cm^2^, respectively.

**Figure 4:**
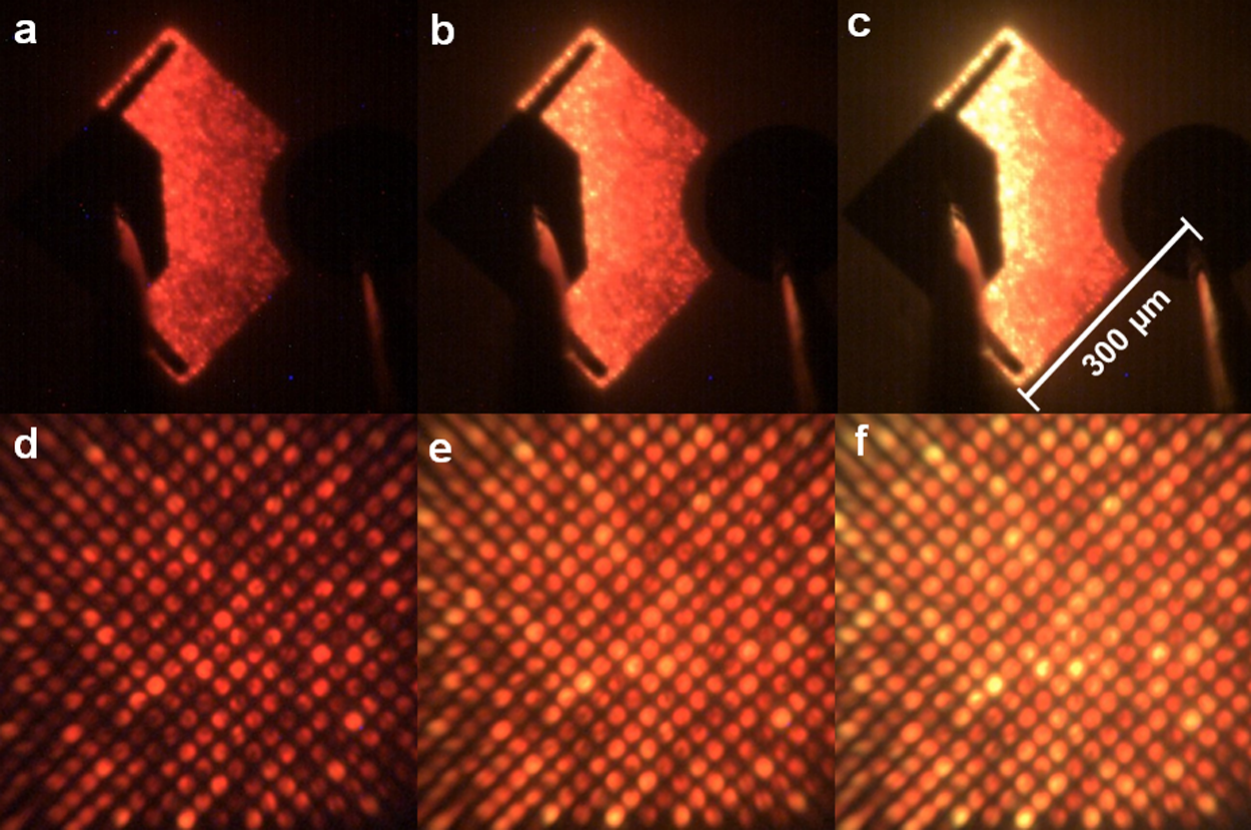
Emission images of the μLED array device taken using an optical microscopy system as a function of injection current density (4, 8, and 12 A/cm^2^) under (a–c) a low magnification and (d–f) a high magnification, respectively.

In order to demonstrate emitting μLED pixels, optical microscopy images have been taken using a micro-EL measurement system where emissions are collected through two objective lenses (one 10× magnification lens with NA = 0.28 and another 50× magnification lens with NA = 0.43). Figure 4a–c displays the emission images of our μLED array chip taken under 4, 8, and 12 A/cm^2^ current density, respectively, while Figure 4d–f provide their corresponding emission images taken under a high magnification, showing strong red emissions from individual 2 μm μLED pixels even under low current densities. It is worth mentioning that such low current densities used for the operation of our μLEDs are lower than a typical current density (22 A/cm^2^) for the operation of a conventional broad area LED.

**Figure 5:**
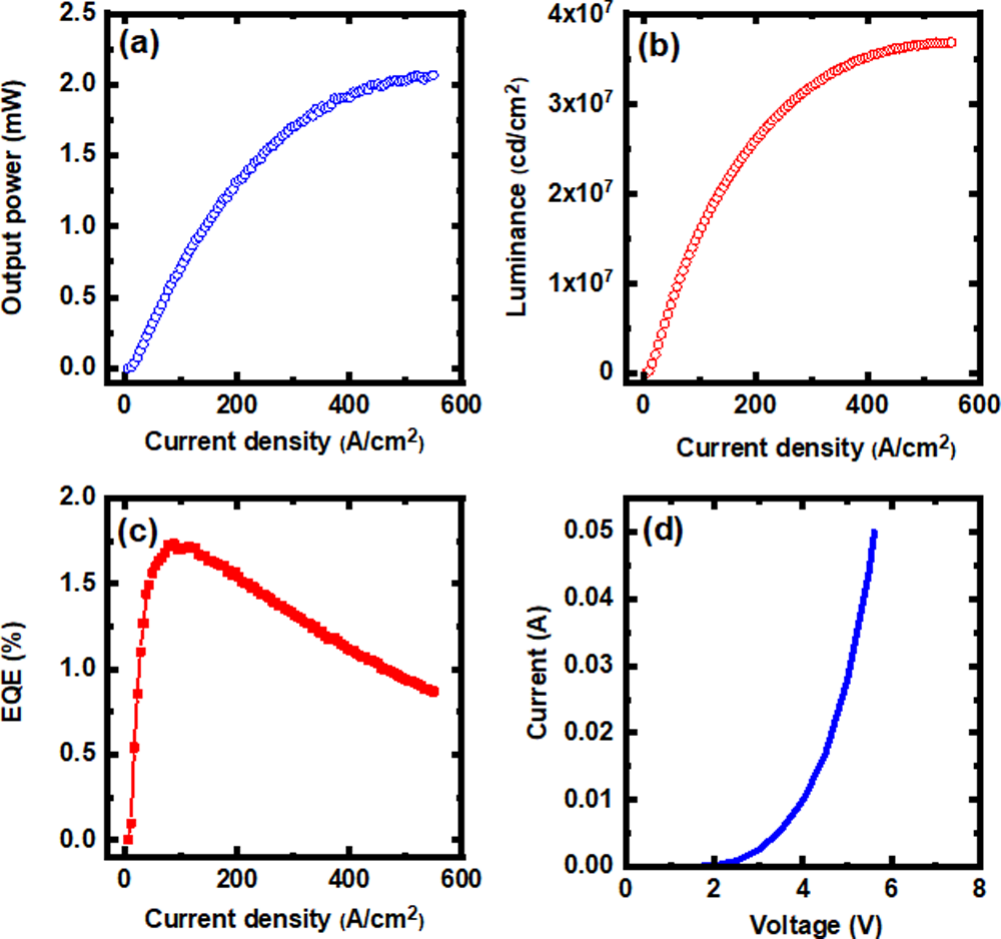
(a) Output power, (b) luminance, (c) EQE, and (d) current–voltage characteristics of the μLED array device (i.e., LED A).

Both light output power and luminous flux have been measured on the bare-chip LEDs bonded on TO5-headers in CW mode using a LCS-100 integrating sphere equipped with a CCD APRAR spectrometer. Figure 5a–c shows the output power, luminance, and EQE of the μLED array device (i.e., LED A) as a function of injection current density. This demonstrates that the output power and luminescence increase monolithically with increasing current density up to 450 A/cm^2^ and that a high luminance of 3.5 × 10^7^ cd/m^2^ has been achieved. The peak EQE is about 1.75%. It is worth highlighting that although there are not any heat-sink components used, our ultrasmall μLEDs can still sustain a high current density of above 450 A/cm^2^, also confirming the high crystal quality of our μLED array sample achieved by our selective epitaxy growth approach. Figure 5d displays the typical *I*–*V* characteristics of LED A measured as a function of bias, which is similar to that of the LED B device. This also shows the good electrical property of our μLED array device.

## 3 Conclusions

In summary, we are proposing to employ a selective epitaxy growth approach on a microhole patterned template to significantly enhance strain relaxation, allowing us to not only obtain the natural formation of regularly arrayed μLEDs but also achieve enhanced indium content in the InGaN/GaN MQWs used as an active region for the μLEDs. By means of this approach, we have demonstrated red InGaN-based μLED arrays with a dimension of 2 μm and an interpitch of 1.5 μm. A high luminance of 3.5 × 10^7^ cd/m^2^ and a peak EQE of 1.75% have been achieved for the red μLED array chip in a wafer form without any packaging. In contrast, the standard LED grown under identical conditions but on a standard planar GaN template demonstrates green emission. This means that our approach paves the way for achieving long wavelength InGaN-based μLEDs with ultrasmall dimensions at an elevated growth temperature, at which it is impossible to obtain InGaN-based red LEDs on a standard planar template.

## 4 Experimental Methods

### 4.1 Fabrication of Prepatterned Templates

A 500 nm SiO_2_ dielectric film is deposited on the n-GaN template by a plasma-enhanced chemical vapor deposition (PECVD) technique, followed by employing a standard photolithography and then a dry etching technique to selectively etch the SiO_2_ dielectric layer down to the n-GaN surface by inductively coupled plasma (ICP), forming regularly arrayed microholes with a diameter of 2 μm and an interpitch of 1.5 μm. This pre-patterned template is then used for further selective epitaxy growth. Finally, selective growth only takes place within SiO_2_ microhole regions, naturally forming regularly arrayed μLEDs.

### 4.2 Device Fabrication

Indium-tin-oxide (ITO) is deposited and then undergoes an annealing process in air at 600 °C for 1 min, forming transparent *p*-type contact, while Ti/Al/Ni/Au alloys are prepared as *n*-type contact. Ti/Au alloys are used as *p*-type and *n*-type electrodes. All the characteristics of the LEDs in this paper are conducted on bare chips, namely, no coating, no passivation, no epoxy, or no reflector, which are often employed for obtaining enhanced extraction efficiency.
